# Global burden of vision impairment due to smoking-related cataract: A descriptive study of spatiotemporal trends based on GBD secondary data and projections to 2050

**DOI:** 10.18332/tid/210411

**Published:** 2025-10-31

**Authors:** Yinhe Chen, Yao Li, Pengcheng Hu, Ting Chen, Andrzej Grzybowski, Luoming Huang

**Affiliations:** 1 Department of Ophthalmology, Quanzhou Maternity and Children’s Hospital, Quanzhou City, China; 2 Department of Ophthalmology, The Second Affiliated Hospital of Chongqing Medical University, Chongqing, China; 3 Department of Ophthalmology, The Second Affiliated Hospital of Fujian Medical University, Quanzhou, China; 4 Institute for Research in Ophthalmology, Foundation for Ophthalmology Development, Poznan, Poland; 5 Department of Ophthalmology, University of Warmia and Mazury, Olsztyn, Poland; 6 Department of Ophthalmology and Optometry, The School of Medical Technology and Engineering, Fujian Medical University, Fuzhou, China

**Keywords:** smoking, tobacco, cataract, global burden of disease, vision impairment

## Abstract

**INTRODUCTION:**

Smoking is a major modifiable risk factor for cataract, with strong biological and epidemiological evidence supporting this association. Nevertheless, the global burden and regional variations in vision impairment attributable to smoking-related cataract have not been comprehensively assessed.

**METHODS:**

Using data from the Global Burden of Disease (GBD) 2021 study, we performed a secondary analysis of the global years lived with disability (YLDs) due to smoking-related cataract vision impairment, from 1990 to 2021. Analyses were stratified by age group (30–34 to ≥95 years). Trends were evaluated using age-standardized YLDs rates (ASYLDsR) and estimated annual percentage change (EAPC). Future burden was projected to 2050 using a Bayesian age-period-cohort (BAPC) model.

**RESULTS:**

In 2021, global YLDs due to smoking-related cataract reached 225174 cases (95% uncertainty interval, UI: 151200–325529), representing a 38.9% (95% UI: 35.2–42.5) increase compared with 1990. However, the ASYLDsR declined significantly (EAPC= -1.41%, 95% CI: -1.48 – -1.33). Population growth contributed over 360% to the increase in YLDs in low sociodemographic index (SDI) regions, whereas population aging was the dominant factor in high-SDI regions (contributing 165%). Males accounted for 82% of the global burden. In 2021, the male ASYLDsR was 5.3 times higher than that of females (4.59; 95% UI: 3.24–6.39 vs 0.87; 95% UI: 0.61–1.23, per 100000 population). Countries in Asia bore the heaviest absolute burden, with China and India collectively accounting for 53% of the global total. The BAPC model projected a continued decline in ASYLDsR through 2050, with predicted rates of 2.69 (95% CI: 0.59–4.77) males and 0.42 (95% CI: 0.09–0.82) females.

**CONCLUSIONS:**

Despite a declining age-standardized burden globally, the absolute burden is increasing due to population growth and aging, presenting ongoing challenges, particularly for low- and middle-income countries. Strengthening tobacco control and improving access to cataract surgery are recommended.

## INTRODUCTION

Cataracts constitute a major public health challenge as the leading cause of global blindness. Data from the Global Burden of Disease (GBD) study show that cataracts persistently rank as the primary cause of blindness worldwide, accounting for approximately 15.2 million cases and representing 45% of the global blindness burden^1^. Given that cataract treatment is highly dependent on surgical intervention, it imposes a heavy socioeconomic burden, particularly in regions with scarce healthcare resources^2^. Previous research has identified numerous likelihood factors for cataract, including age, sex, ethnicity, and environmental factors such as ultraviolet radiation exposure and tobacco smoke exposure^3^. Therefore, identifying and proactively controlling modifiable likelihood factors for cataract is imperative.

Smoking represents one of the major global public health threats, causing over 7.5 million deaths annually worldwide^4^. Substantial evidence indicates that tobacco smoke exposure is strongly associated with several blinding eye diseases, including cataract, glaucoma, and age-related macular degeneration^5,6^. Experimental studies have revealed potential mechanisms involving tobacco-induced oxidative stress and subsequent photochemical reactions, leading to lens injury and accelerated cross-linking denaturation of α-crystallin proteins^7^. Epidemiological studies have consistently confirmed the association between smoking and an elevated likelihood of cataract^8-11^. Compared to never smokers, individuals with a history of smoking exhibit a significantly higher prevalence ratio for cataract and a higher rate of undergoing cataract surgery^12^. Furthermore, a large prospective cohort study demonstrated that exposure to tobacco smoke increases the risk of developing cataract; compared to never smokers, individuals exposed during childhood, adolescence, and adulthood exhibited increased risks^9^.

However, existing studies exhibit significant limitations: they lack long-term spatiotemporal dynamic analyses of the years lived with disability (YLDs) burden attributable to smoking-related cataract stratified by the sociodemographic index (SDI); the impact of tobacco control policies on the disease burden has not been systematically quantified, hindering the evaluation of public health intervention effectiveness; and current predictive models fail to integrate the complex interaction effects of population aging and declining smoking rates, limiting accurate projections of future burden.

Leveraging GBD 2021 data, this study quantifies the visual impairment burden from smoking-related cataracts (1990–2021) across geography, SDI, age, and sex, with projections to 2050, to provide deeper insights into the global disease burden.

## METHODS

### Data source

This study is a secondary analysis based on the GBD 2021 database. Data were sourced from the publicly accessible database hosted by the Institute for Health Metrics and Evaluation (IHME) at the University of Washington (https://ghdx.healthdata.org/gbd-2021). This database has undergone institutional ethics review, and prior studies have confirmed it meets criteria for exemption from ethics review^13^. The GBD 2021 study integrates multiple heterogeneous data sources – including epidemiological surveys, disease registries, electronic health records, and scientific literature – and employs Bayesian meta-regression tools, particularly DisMod-MR 2.1^6^, to impute and smooth estimates across locations and over time. This approach robustly handles missing and sparse data, producing complete and consistent estimates of mortality, morbidity, and health loss globally.

### Definitions and stratification

Smokers were defined as current or former daily or non-daily users of any smoked tobacco product (including cigarettes, kreteks, pipes, waterpipe, cigars, bidis, and other traditional smoked products). Users of smokeless tobacco, e-cigarettes, and heated tobacco products were excluded. Cataract was defined according to the International Classification of Diseases, Tenth Revision (ICD-10) codes (H25-H26.9, H28-H28.8) as a pathological state characterized by abnormal protein aggregation leading to lens opacity and visual impairment^14^. Countries and territories were stratified using the SDI^15^. The SDI is a composite measure of lag-distributed income per capita, average education level in the population aged ≥15 years, and the total fertility rate under the age of 25 years. Based on the SDI, 204 countries and territories were categorized into five quintiles: low, low-middle, middle, high-middle, and high SDI.

### Data extraction and metrics

From the GBD 2021 database, we extracted the following metrics related to the burden of smoking-related cataract for the period 1990–2021, at the global level, by SDI quintile, and for 204 countries and territories: 1) Years lived with disability (YLDs), quantifying the burden of disability caused by the disease; and 2) Age-standardized YLDs rate (ASYLDsR), expressed per 100000 population, to eliminate the influence of population age structure. Data were inclusive of all sexes and age groups from 30 to ≥95 years. No additional exclusion criteria were applied. Age and sex were considered as potential confounders in the modeling process through stratification and standardization.

### Statistical analysis

To quantify the spatiotemporal trends in the global burden of smoking-related cataract from 1990 to 2021, we used the ASYLDsR as the core metric and assessed its temporal dynamics using the EAPC. The EAPC was calculated based on a linear regression model applied to the natural logarithm of the ASYLDsR against calendar year: y = α + βx + ε, where y = ln (ASYLDsR), x is the calendar year, α is the intercept, β is the slope, and ε is the error term. The regression coefficient β was transformed using the formula: EAPC = 100 × [exp(β) – 1]. The direction of the trend was determined based on the value of β and its 95% confidence interval (CI): If β >0 and the lower limit of the 95% CI >0, the burden was considered to have an increasing trend. If β <0 and the upper limit of the 95% CI <0, the burden was considered to have a decreasing trend. If the 95% CI included 0, the trend was considered stable^16^. To visually present the temporal evolution of the disease burden, comparative analyses were performed using data from 1990 and 2021. Descriptive subgroup analyses were conducted to examine burden distribution patterns across sex, age groups (30–34 to ≥95 years), geographical regions, and SDI quintiles. The linearity assumption of the EAPC model was verified through visual inspection of log-linear plots and residual analysis. The decomposition analysis employed the classical Das Gupta method^17^ to attribute the change in YLDs from 1990 to 2021 to three factors: change in population size (solely due to change in number of population), change in population age structure (solely due to aging), and the relative contribution of epidemiological change to vision impairment-related YLDs caused by smoking-associated cataract. The projection of the future disease burden utilized the BAPC model. This model integrates age, period, and birth cohort effects to analyze the complex interactions of demographic factors on disease burden events. Its Bayesian framework effectively handles complex data structures and uncertainty^18^. All analyses were conducted in R Studio (version 4.3.3). Key metrics are reported with their corresponding 95% uncertainty intervals (UI). UIs are used throughout the GBD study to reflect stochastic uncertainty from multiple modeling steps, while CIs are derived from regression models. UIs are presented for burden estimates, and CIs for trend statistics.

## RESULTS

### Global burden trends

**Figure 1:**
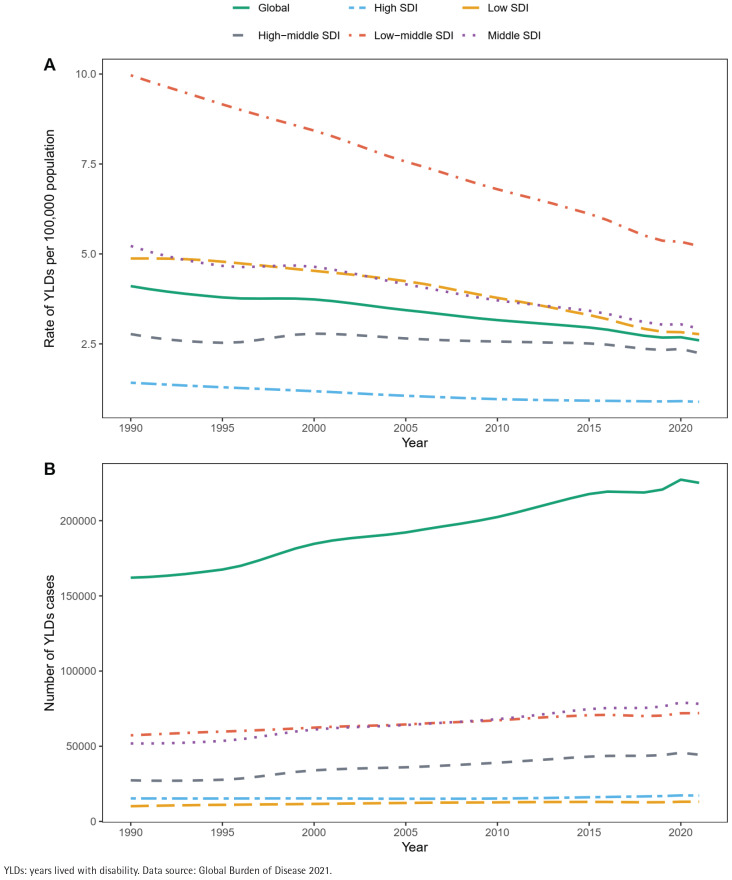
Trends in age-standardized YLDs rates (A) and number of YLDs cases (B) for smoking-related cataract globally and by SDI quintile, 1990–2021. Trends were smoothed using LOESS regression for visual representation. Countries/regions by SDI quintile: Low (34), Low-middle (48), Middle (41), High-middle (48), High (33)

**Figure 2:**
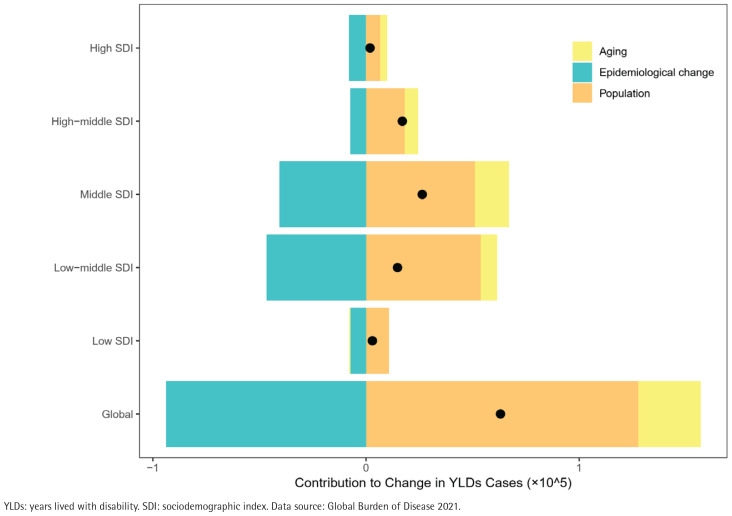
Changes in smoking-related cataract YLDs attributable to population-level determinants (population growth, aging, and epidemiological change) from 1990 to 2021 at the global level and by SDI quintile. The black dot represents the overall value of change contributed by all 3 components. Countries/regions by SDI quintile: Low (34), Low-middle (48), Middle (41), High-middle (48), High (33)

In 2021, the global YLDs attributable to smoking-related cataract reached 225174 cases (95% UI: 151200–325529), representing a 38.9% increase compared with 1990 (162083 cases; 95% UI: 108854–230787). Concurrently, the ASYLDsR significantly declined, decreasing from 8.62 per 100000 population (95% UI: 5.81–12.26) in 1990 to 4.11 per 100000 (95% UI: 2.94–5.63) in 2021, with an EAPC of -1.41% (95% CI: -1.48 – -1.33) (Table 1 and Figure 1). Decomposition analysis revealed significant disparities in the drivers of YLDs growth: population growth (+202.47%) and population aging (+46.31%) acted as positive drivers, while epidemiological changes (-148.78%) exerted a negative effect (Figure 2, and Supplementary file Table 1). Population growth contributed most substantially in low-middle SDI (+364.30%) and low SDI (+363.47%) regions. Population aging had the most pronounced impact in high SDI regions (+165.55%), whereas the negative effect of epidemiological changes was most prominent in high SDI regions (-417.03%).

**Table 1:** Estimated YLDs cases, age-standardized YLDs rate per 100000 population, and temporal trends of vision impairment due to smoking-related cataract, by sex, age group, SDI, and region, 1990–2021

	*1990*		*2021*		*1990 to 2021*
	*Cases (95% UI)*	*Age standardized YLDs rate per 100000 population* *(95% UI)*	*Cases (95% UI)*	*Age standardized YLDs rate per 100000 population* *(95% UI)*	*EAPC (95% CI)*
**Global**	162083 (115037–221809)	4.11 (2.94–5.63)	225174 (160701–315105)	2.6 (1.85–3.63)	-1.41 (-1.48 – -1.33)
**Sex**					
Male	131529 (93031–180121)	7.38 (5.3–10.1)	184884 (130572–258505)	4.59 (3.24–6.39)	-1.46 (-1.52 – -1.4)
Female	30554 (21971–41654)	1.45 (1.05–1.97)	40290 (28391–56799)	0.87 (0.61–1.23)	-1.6 (-1.77 – -1.44)
**Age** (years)					
30–34	2861 (1660–4168)	0.74 (0.43–1.08)	2006 (1157–2978)	0.33 (0.19–0.49)	-2.84 (-3.03 – -2.64)
35–39	4802 (2938–7068)	1.36 (0.83–2.01)	3690 (2252–5405)	0.66 (0.4–0.96)	-2.56 (-2.7 – -2.43)
40–44	6794 (4347–9889)	2.37 (1.52–3.45)	6151 (3923–9167)	1.23 (0.78–1.83)	-2.29 (-2.36 – -2.22)
45–49	9658 (6473–14024)	4.16 (2.79–6.04)	10591 (6901–15529)	2.24 (1.46–3.28)	-2.04 (-2.09 – -1.99)
50–54	14068 (9365–20729)	6.62 (4.41–9.75)	17082 (11418–25366)	3.84 (2.57–5.7)	-1.79 (-1.85 – -1.72)
55–59	18191 (12413–26501)	9.82 (6.7–14.31)	24548 (16380–35922)	6.2 (4.14–9.08)	-1.4 (-1.46 – -1.35)
60–64	23734 (15224–33789)	14.78 (9.48–21.04)	30138 (19598–43467)	9.42 (6.12–13.58)	-1.43 (-1.53 – -1.33)
65–69	25198 (17003–35397)	20.39 (13.76–28.64)	36922 (24621–53341)	13.39 (8.93–19.34)	-1.19 (-1.32 – -1.06)
70–74	22179 (15458–31058)	26.2 (18.26–36.69)	34966 (23739–49783)	16.99 (11.53–24.19)	-1.08 (-1.22 – -0.95)
75–79	17112 (11916–23514)	27.8 (19.36–38.2)	27334 (19256–39544)	20.73 (14.6–29.98)	-0.89 (-0.98 – -0.81)
80–84	10656 (7378–15027)	30.12 (20.86–42.48)	17795 (12391–25193)	20.32 (14.15–28.76)	-1.19 (-1.34 – -1.03)
85–89	5080 (3487–7136)	33.62 (23.08–47.23)	9747 (6733–13785)	21.32 (14.73–30.15)	-1.59 (-1.72 – -1.47)
90–94	1433 (970–2035)	33.44 (22.64–47.49)	3380 (2277–4821)	18.9 (12.73–26.95)	-2.04 (-2.19 – -1.89)
≥95	318 (222–452)	31.27 (21.81–44.43)	823 (554–1228)	15.11 (10.17–22.53)	-2.41 (-2.56 – -2.26)
**SDI region**					
High	15327 (10710–21854)	1.42 (0.99–2.03)	17228 (11581–25168)	0.89 (0.6–1.31)	-1.6 (-1.7 – -1.5)
High-middle	27352 (19451–37917)	2.77 (1.96–3.83)	44388 (31100–63253)	2.25 (1.58–3.19)	-0.41 (-0.55 – -0.27)
Middle	51857 (36897–71637)	5.22 (3.72–7.17)	78234 (55296–110583)	2.93 (2.08–4.13)	-1.77 (-1.88 – -1.67)
Low-middle	57281 (40924–78507)	9.97 (7.16–13.55)	72051 (51674–99415)	5.22 (3.72–7.18)	-2.08 (-2.14 – -2.02)
Low	10157 (7093–13910)	4.87 (3.44–6.55)	13144 (9350–18245)	2.77 (1.97–3.83)	-1.9 (-2.08 – -1.73)
**Region**					
Andean Latin America	599 (395–879)	3.05 (2.01–4.5)	1013 (638–1558)	1.73 (1.09–2.69)	-2.28 (-2.44 – -2.12)
Australasia	239 (163–338)	1.04 (0.7–1.48)	306 (200–459)	0.62 (0.41–0.94)	-1.55 (-1.6 – -1.5)
Caribbean	681 (466–999)	2.64 (1.78–3.88)	738 (485–1102)	1.37 (0.9–2.04)	-2.23 (-2.32 – -2.14)
Central Asia	1151 (786–1636)	2.41 (1.66–3.43)	1772 (1208–2507)	2.13 (1.45–3.01)	-0.2 (-0.37 – -0.03)
Central Europe	1938 (1331–2766)	1.31 (0.9–1.86)	1701 (1145–2452)	0.83 (0.56–1.2)	-1.58 (-1.63 – -1.54)
Central Latin America	2547 (1774–3552)	3.19 (2.24–4.47)	2783 (1889–3992)	1.12 (0.77–1.62)	-3.62 (-3.72 – -3.52)
Central Sub-Saharan Africa	119 (78–168)	0.52 (0.35–0.72)	203 (138–288)	0.34 (0.23–0.48)	-1.16 (-1.3 – -1.01)
East Asia	31913 (22457–44321)	3.82 (2.7–5.26)	60301 (41336–85030)	2.74 (1.88–3.87)	-0.6 (-0.83 – -0.37)
Eastern Europe	2996 (2127–4272)	1.09 (0.77–1.54)	3392 (2356–4814)	1.01 (0.71–1.44)	-0.2 (-0.48–0.09)
Eastern Sub-Saharan Africa	1792 (1246–2440)	2.4 (1.68–3.29)	2403 (1677–3361)	1.36 (0.95–1.9)	-1.81 (-1.9 – -1.73)
High-income Asia Pacific	2570 (1804–3629)	1.27 (0.9–1.8)	2732 (1854–3975)	0.67 (0.46–0.96)	-2.2 (-2.27 – -2.14)
High-income North America	4656 (3171–6753)	1.36 (0.93–1.97)	4987 (3300–7621)	0.8 (0.53–1.21)	-1.87 (-2.04 – -1.69)
North Africa and Middle East	9737 (6766–13559)	6.06 (4.19–8.53)	15315 (10759–21665)	3.43 (2.4–4.82)	-1.94 (-2 – -1.89)
Oceania	187 (125–264)	5.83 (3.86–8.17)	372 (249–554)	4.46 (3.02–6.63)	-0.91 (-1 – -0.82)
South Asia	62501 (44760–85731)	11.76 (8.4–15.95)	79609 (56689–110779)	5.64 (4.01–7.85)	-2.38 (-2.47 – -2.3)
Southeast Asia	19522 (13922–26799)	7.98 (5.68–10.92)	27758 (19572–38613)	4.28 (3.03–5.92)	-2.23 (-2.34 – -2.12)
Southern Latin America	783 (527–1146)	1.68 (1.13–2.46)	870 (563–1295)	1.04 (0.67–1.53)	-1.54 (-1.62 – -1.47)
Southern SubSaharan Africa	1386 (965–1976)	5.1 (3.53–7.18)	1001 (678–1389)	1.67 (1.14–2.31)	-3.89 (-4.09– -3.68)
Tropical Latin America	5457 (3691–7701)	6.12 (4.13–8.67)	6408 (4125–9643)	2.5 (1.62–3.77)	-2.67 (-2.98 – -2.36)
Western Europe	9949 (6814–14295)	1.84 (1.26–2.61)	8939 (5889–12969)	1.08 (0.72–1.61)	-1.73 (-1.79 – -1.67)
Western Sub-Saharan Africa	1358 (930–1908)	1.57 (1.08–2.2)	2570 (1728–3656)	1.26 (0.85–1.82)	-0.87 (-1.11 – -0.63)

YLDs: years lived with disability. UI: uncertainty intervals. CI: confidence interval. SDI: sociodemographic index. EAPC: estimated annual percentage change. Countries/regions by SDI quintile: Low (34), Low-middle (48), Middle (41), High-middle (48), High (33). Data source: Global Burden of Disease 2021.

### Sex and age stratification

**Figure 3:**
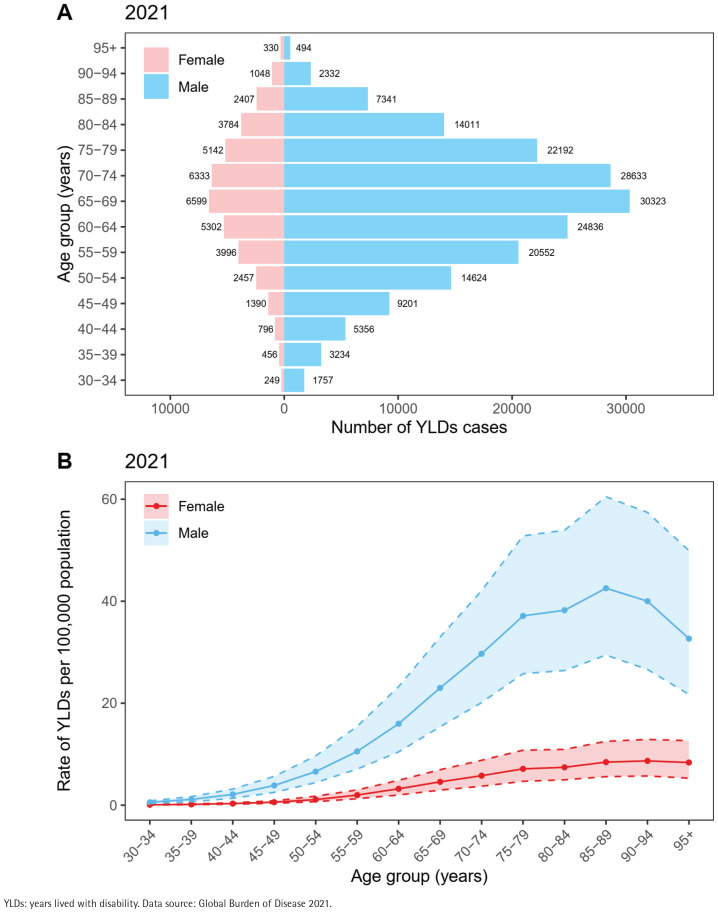
Number of YLDs cases (A) and age-standardized YLDs rates (B) for smoking-related cataract by age group and sex, 2021. Trends were smoothed using LOESS regression for visual representation. The shaded areas represent the 95% uncertainty intervals. Trends were smoothed using LOESS regression for visual representation.

The number of cases was consistently higher among males than females. Male YLDs increased from 131529 cases (95% UI: 88071–187121) in 1990 to 184884 cases (95% UI: 124009–266453) in 2021. The male ASYLDsR decreased from 7.38 per 100000 (95% UI: 5.3–10.1) to 4.59 per 100000 (95% UI: 3.24–6.39) over this period (EAPC= -1.46%; 95% CI: -1.52 – -1.40). Female YLDs increased from 30554 cases (95% UI: 19852–44402) in 1990 to 40290 cases (95% UI: 25965–60938) in 2021, with the ASYLDsR declining from 1.45 per 100000 (95% UI: 1.05–1.97) to 0.87 per 100000 (95% UI: 0.61–1.23) (EAPC= -1.60%, 95% CI: -1.77 – -1.44) (Table 1, and Supplementary file Figure 1). Age distribution analysis showed that both the number of cases and the age-standardized rate increased with age. The 65–69 years age group bore the heaviest burden in 2021 (36922 cases; 95% UI: 24621–53341 and ASYLDsR=13.39 per 100000; 95% UI: 8.93–19.34), exhibiting an annual rate reduction of -1.19% (95% CI: -1.32 – -1.06). While the 30–34 years age group had the largest absolute number of cases, it also showed the steepest decline in ASYLDsR, falling from 0.74 per 100000 (95% UI: 0.43–1.08) in 1990 to 0.33 per 100000 (95% UI: 0.19–0.49) in 2021 (EAPC= -2.84%; 95% CI: -3.03 – -2.64) (Table 1 and Figure 3).

### SDI and geographical heterogeneity

High SDI regions had the lowest ASYLDsR in 2021 (0.89 per 100000; 95% UI: 0.6–1.31), significantly lower than in 1990 (1.42 per 100000; 95% UI: 0.99–2.03) (EAPC= -1.60%; 95% CI: -1.70 – -1.50). Conversely, low-middle SDI regions exhibited the highest ASYLDsR in 2021 (5.22 per 100000; 95% UI: 3.72–7.18) but also the largest rate reduction (EAPC = -2.08%; 95% CI: -2.14 – -2.02) (Table 1 and Figure 1). SDI level was inversely associated with ASYLDsR (Supplementary file Figure 2). Country-level analysis revealed that high-income countries experienced concurrent decreases in both YLDs cases and ASYLDsR. In contrast, low- and middle-income countries saw an increase in YLDs cases alongside a decrease in ASYLDsR (Supplementary file Figures 3 and 4). The burden was particularly pronounced in Asian countries, with China (60057 cases) and India (59155 cases) collectively contributing 52.9% of the global YLDs total in 2021 (Supplementary file Table 1).

### Burden projection

Projections from the BAPC model (Supplementary file Figure 5) indicate that the global ASYLDsR for smoking-related cataract is expected to continue declining between 2022 and 2050.

## DISCUSSION

Based on the GBD 2021 study, this research systematically evaluated the spatiotemporal evolution of the global burden of vision impairment due to smoking-related cataract from 1990 to 2021. The results indicate that although the global ASYLDsR for smoking-related cataract showed a significant declining trend, the number of YLDs cases increased. This phenomenon is primarily driven by population growth and aging. However, epidemiological changes related to tobacco exposure – including declining smoking prevalence, increased smoking cessation rates, and improved access to cataract surgery – have partially offset this increase^4,19^. This finding underscores the profound impact of global demographic transition on disease burden, demonstrating that even with improvements in age-standardized rates, the absolute disease burden may still rise due to population expansion and intensifying aging^15^.

Demographic decomposition analysis further revealed regional heterogeneity. Population growth contributed a substantial 364% to the increase in YLDs in low-middle SDI regions, reflecting their early-stage demographic transition characteristics. In contrast, the significant contribution of population aging and the strong negative effect of epidemiological changes in high SDI regions confirm the transformative role of tobacco control legislation, universal health coverage, and high-quality ophthalmic services. These factors have successfully shifted smoking-related cataract from a highly disabling condition towards a manageable disease^4^. Furthermore, low-SDI countries, currently characterized by higher fertility rates and younger population structures, are transitioning from high fertility toward rapid aging^20^. Within this context, and given persistent inadequacies in healthcare resources^21^, the synergistic progress in tobacco exposure management and vision health services calls for sustained attention.

Sex and age disparities highlight heterogeneity in disease distribution. The burden of YLDs remained consistently higher among males than females; in 2021, the male ASYLDsR was 5.3 times higher than the female rate, and males accounted for 82% of global cases. This disparity closely associates with the global epidemiological pattern of significantly higher smoking prevalence among males compared to females^4^. A meta-analysis by Beltrán-Zambrano et al.^22^ confirmed that current smokers have a significantly increased risk of cataract, with the strongest association observed for nuclear cataract. Biologically, oxidative stressors in tobacco smoke, such as cadmium ions, accelerate the cross-linking denaturation of α-crystallin proteins in the lens^23^. This process may be exacerbated in males due to age-related declines in the lens’s antioxidant capacity^23^. It is noteworthy that the age-standardized rates declined continuously for both sexes, reflecting the combined success of global tobacco control policies and ophthalmic interventions^24^.

Age-stratified analysis revealed that the YLDs burden increased with age, peaking in the 65–69 years group, while the 30–34 years group exhibited the steepest decline in ASYLDsR. This trend aligns with the age-related nature of cataract, where oxidative damage to lens proteins accumulates over time, a process accelerated by smoking. Younger populations, with shorter cumulative exposure to tobacco and greater responsiveness to cessation interventions, experienced more significant declines in age-standardized rates^4^. This suggests that early interventions targeting younger populations, such as smoking cessation education, could effectively delay or prevent the onset of smoking-related cataract^25^. However, increases in youth smoking prevalence in low- and middle-income countries, along with the growing use of novel tobacco products such as e-cigarettes, could contribute to an elevated likelihood of early-onset cataract^20^, suggesting a need for targeted prevention approaches.

Disparities across SDI regions reveal global inequities in eye health. The age-standardized YLDs rate in low SDI regions was 3.1 times higher than in high SDI regions, reflecting the structural roots of imbalanced healthcare resource allocation. High SDI countries, through comprehensive tobacco control policies (such as Australia’s plain packaging legislation and the UK’s universal smoking cessation services) and efficient cataract surgical networks, have achieved a ‘low-level stable’ status for smoking-related cataract^26^. Conversely, low SDI regions face severe challenges in their prevention and control systems due to scarce healthcare resources leading to low cataract surgical coverage, weak implementation of tobacco control policies^24,27^, and persistently high smoking prevalence^4^. Furthermore, the decline in age-standardized rates was faster in low SDI regions compared to high SDI regions. This phenomenon may stem from the greater marginal benefits of interventions in settings with a high baseline burden^26^. Decomposition analysis further corroborates the mechanisms of regional heterogeneity: population expansion was the primary driver of YLDs growth in low SDI regions, while aging dominated in high SDI regions. This disparity suggests that regionally tailored strategies may be needed: low-SDI regions may need to prioritize strengthening tobacco control legislation and expanding primary eye care network coverage, whereas high-SDI regions should focus on optimizing cataract screening pathways and early surgical intervention for their aging populations^28^.

Heterogeneity at the national level highlights the complex distribution of the disease burden. Asian countries bear a high absolute burden due to their large population bases, with China and India collectively contributing 53% of global YLDs. Although both countries exhibited declining ASYLDsR, accelerating population aging is driving continued growth in the absolute burden. China faces unique challenges: the smoking prevalence among individuals aged ≥40 years was 27.2% in 2019–2020, the male smoking rate was 21 times that of females, and per capita tobacco consumption exceeded the global average^29^; simultaneously, cataract surgical rates in rural areas remain significantly lower than those in high-income countries^30^. In contrast, high-income countries have achieved rapid reductions in disease burden through stringent tobacco control regulations (e.g. smoke-free public places, tobacco taxation) and highly efficient surgical service systems^31,32^.

### Limitations

This study has several limitations. First, as the GBD study relies on modeled estimates, the quality and coverage of primary data in some countries – particularly in low SDI regions – may be limited, which could introduce estimation bias. Second, the calculation of YLDs is based on population attributable fractions (PAF), the accuracy of which depends on the quality of smoking exposure data and the relative risk estimates for the association between smoking and cataract. Although GBD uses standardized Bayesian models to quantify uncertainty, some residual uncertainty remains. Third, variations in cataract diagnostic criteria across regions and over time may affect consistency in estimates. While GBD attempts to harmonize these differences through disease modeling, cross-regional comparability may still be compromised. Fourth, despite rigorous adjustment in GBD methods, residual confounding may persist. Fifth, underreporting of smoking – especially among women and youth – may lead to underestimation of the burden. Sixth, emerging trends such as e-cigarette use were not included due to a lack of sufficient long-term data. Finally, the ‘epidemiological change’ component in the decomposition analysis represents a residual term derived indirectly, encompassing both residual confounding and other non-demographic factors such as changes in diagnostic coding practices; thus, this component requires cautious interpretation. Moreover, the findings of this study reflect population-level associations and should not be interpreted as establishing causality.

## CONCLUSIONS

The global burden of vision impairment due to smoking-related cataract remains a significant public health concern. Despite the declining trend in the global ASYLDsR, the absolute number of YLDs cases has increased, underscoring the underlying drivers of population growth and aging. The disease burden continues to be disproportionately heavy in low- and middle-income countries.

## Supplementary Material



## Data Availability

The data supporting this research are available from the following source: http://ghdx.healthdata.org
