# Supplementary material for: Global burden of vision impairment due to smoking-related cataract: A descriptive study of spatiotemporal trends based on GBD secondary data and projections to 2050
Source: Tob Induc Dis. 2025 Oct 31;23:10.18332/tid/210411. doi: 10.18332/tid/210411 (PMC12579110; doi:10.18332/tid/210411)
Supplement: Supplementary file 1 [file TID-23-166-s1.pdf]

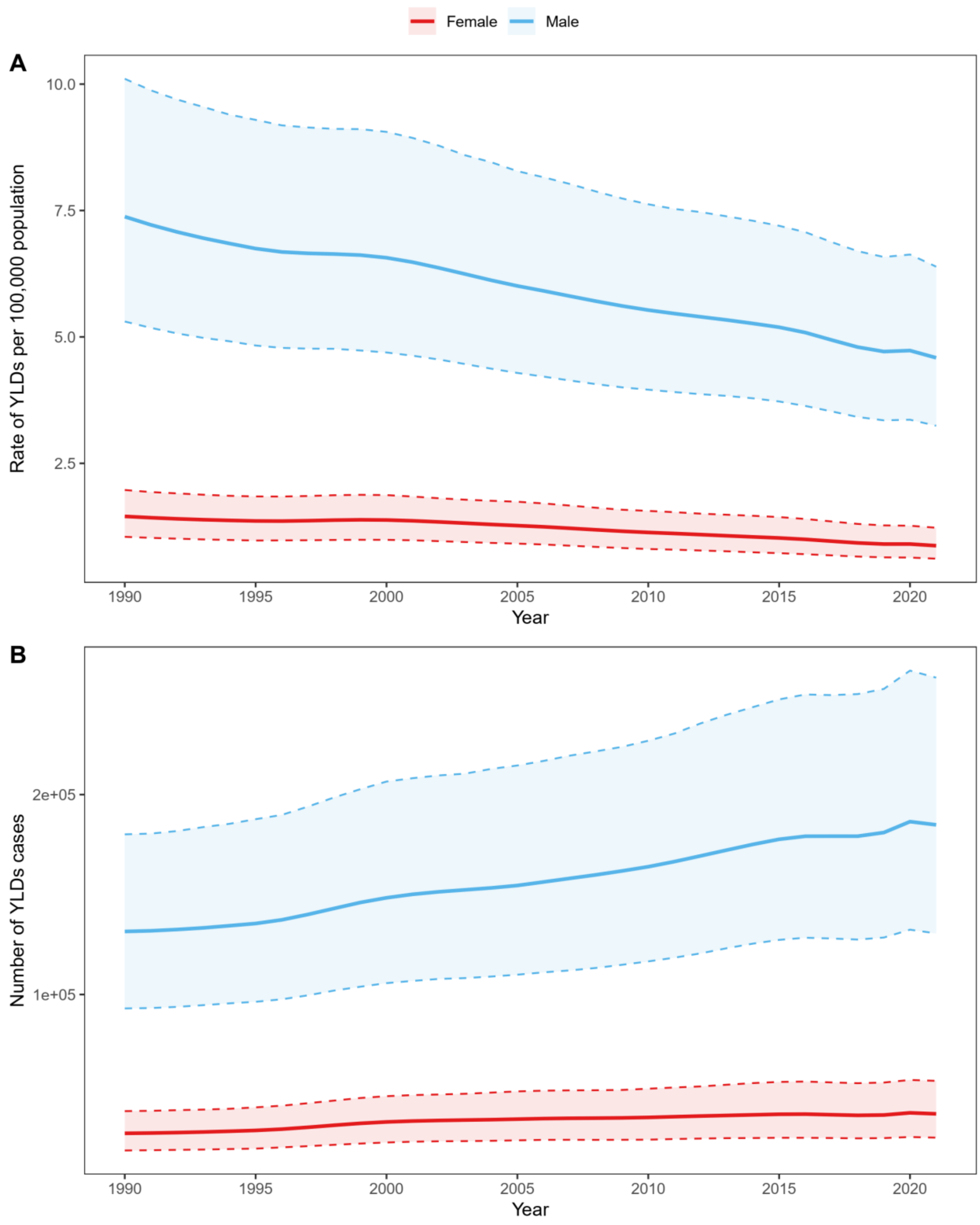

Supplementary file Figure 1. Trends in age-standardized YLD rates (A) and number of YLDs cases (B) for smoking-related cataract in males and females, 1990–2021. The shaded areas represent the 95% uncertainty intervals. Trends were smoothed using LOESS regression for visual representation. YLDs: years lived with disability. Data source: Global Burden of Disease 2021.

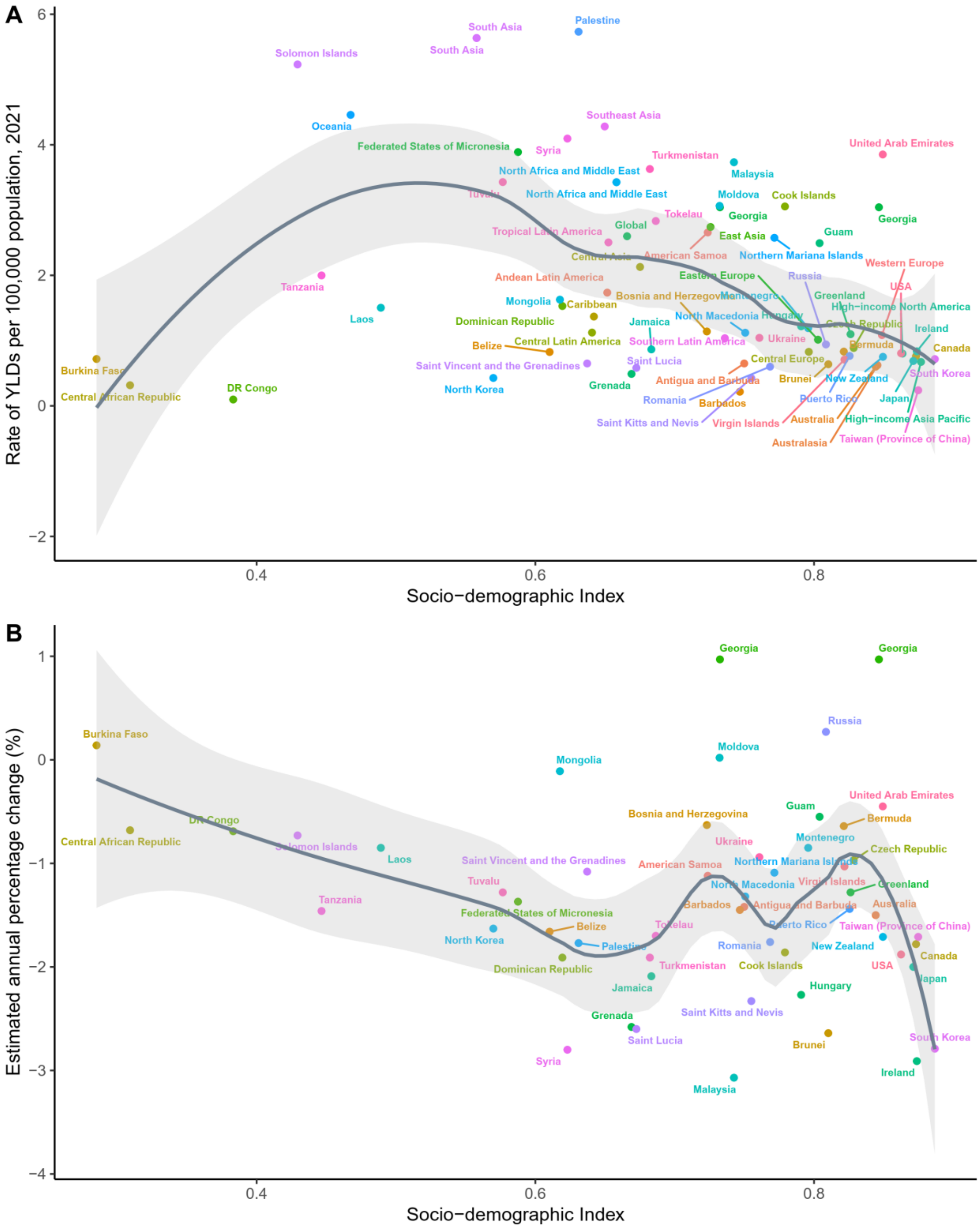

Supplementary file Figure 2. Correlation analysis between SDI and age-standardized YLDs rates (A) and their estimated annual percentage change (B) globally in 2021. The shaded areas represent the 95% confidence intervals. YLDs: years lived with disability; SDI: socio-demographic index. SDI is a composite measure of income per capita, educational attainment, and fertility rate. Higher SDI indicates higher sociodemographic development. Data source: Global Burden of Disease 2021.

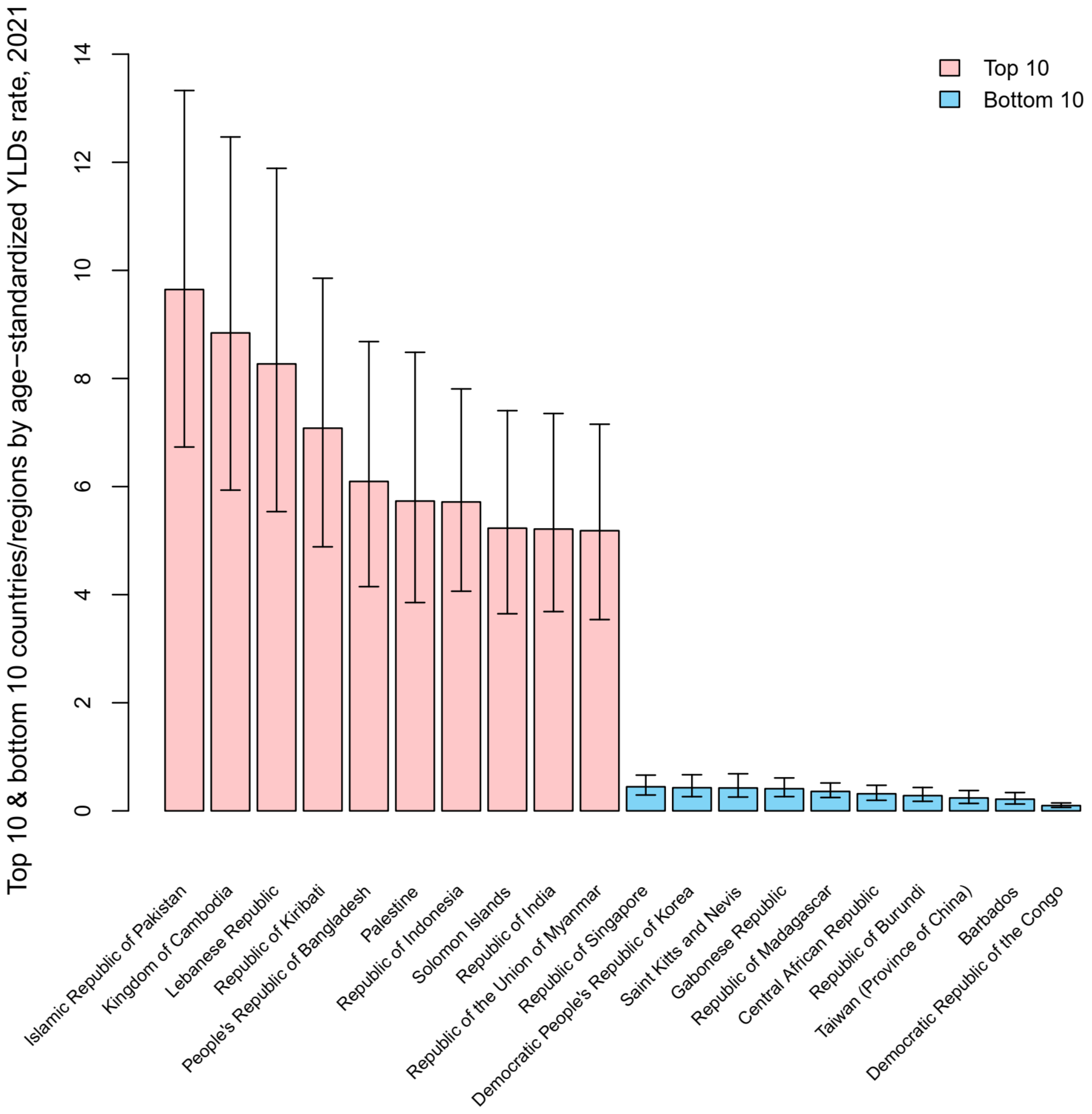

Supplementary file Figure 3. Highest and Lowest age-standardized YLDs Rates of Smoking-Related cataract by Countries/Regions (2021). Error bars represent the 95% uncertainty intervals. YLDs: years lived with disability. Data source: Global Burden of Disease Study 2021.

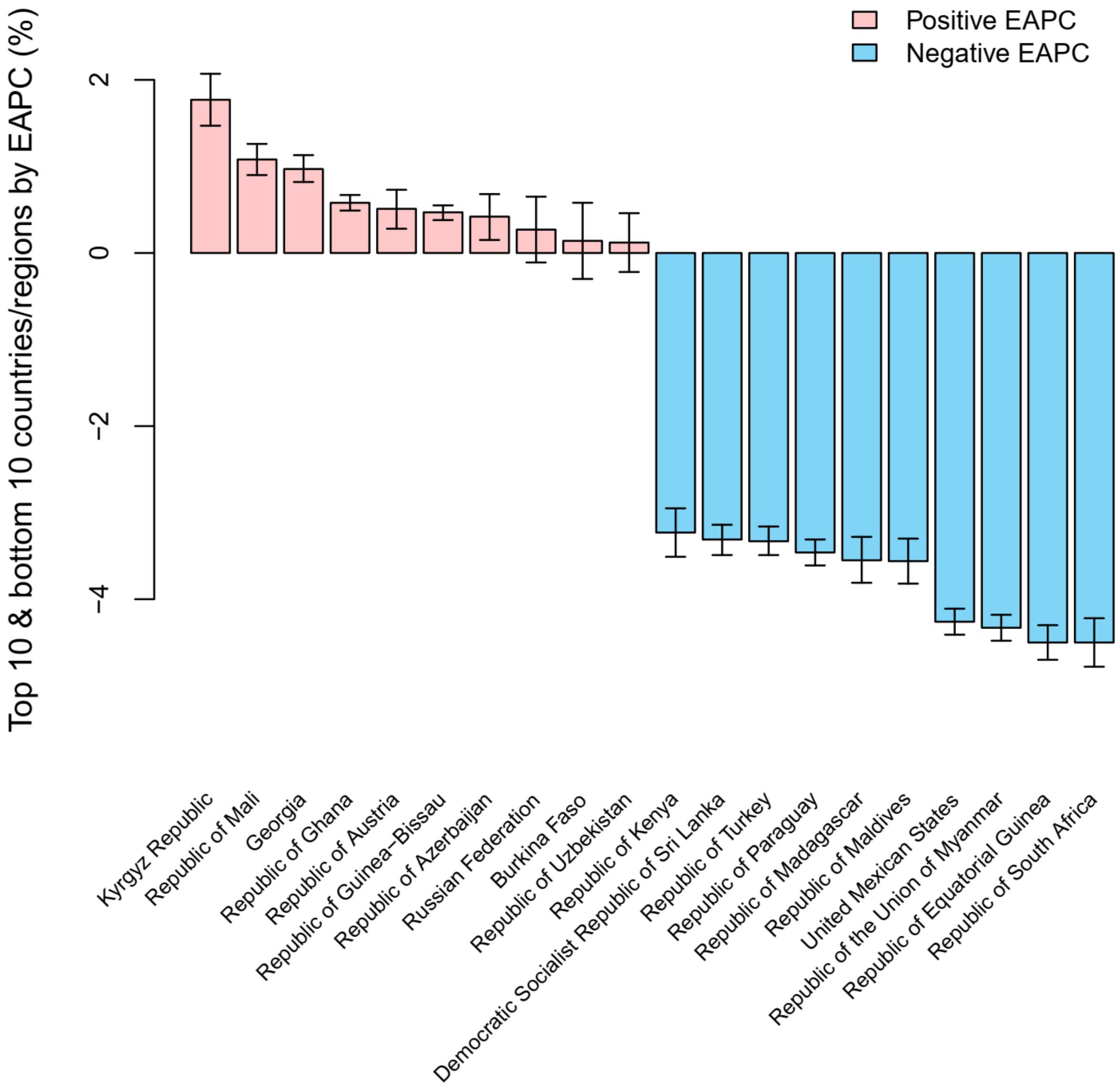

Supplementary file Figure 4. Highest and Lowest age-standardized YLDs Rates of Smoking-Related cataract by Countries/Regions (2021). Error bars represent the 95% uncertainty intervals. YLDs: years lived with disability; EAPC: estimated annual percentage change. Data source: Global Burden of Disease Study 2021.

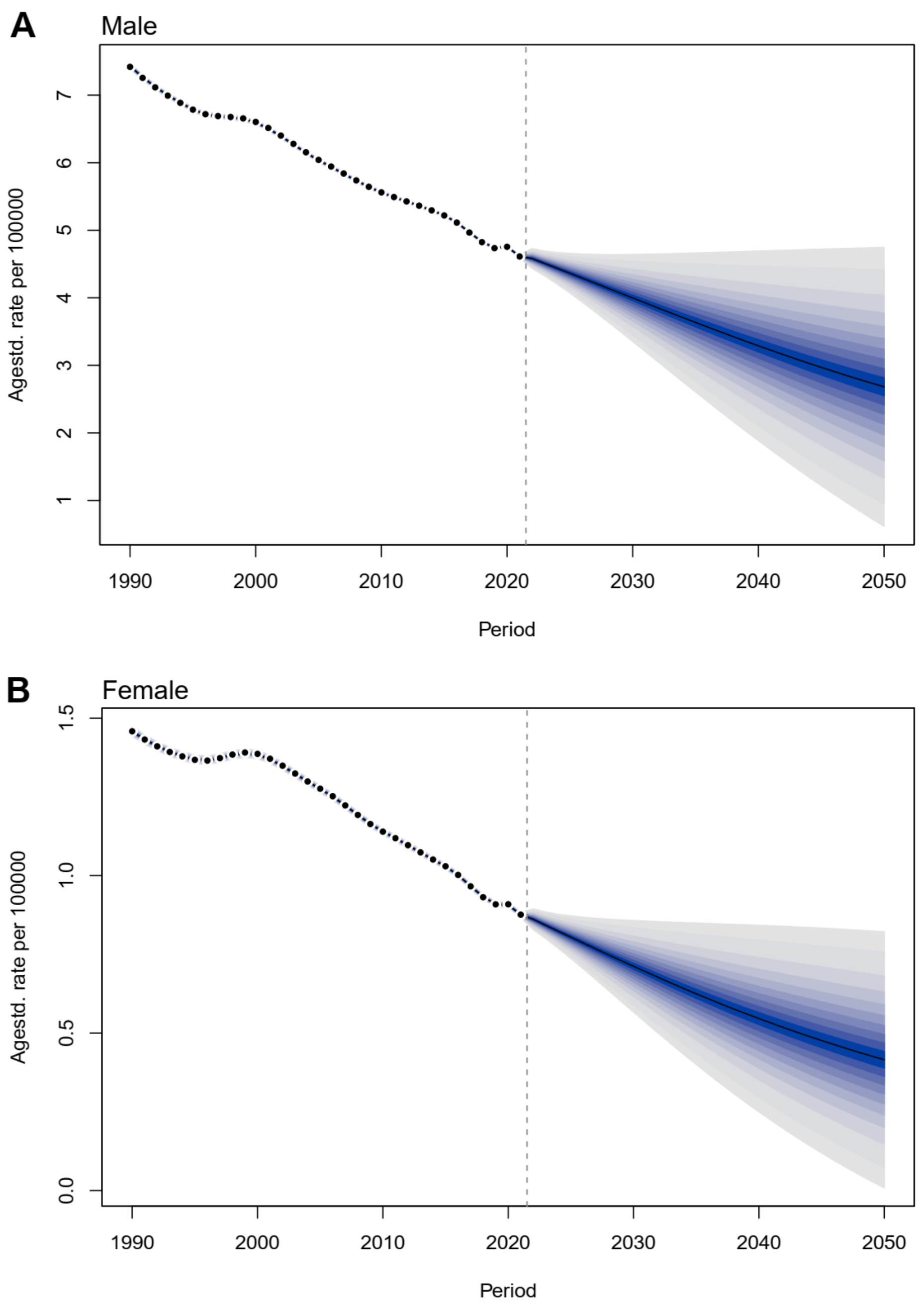

Supplementary file Figure 5. Historical trends and projections to 2050 in age-standardized YLDs rates for smoking-related cataract, estimated with the Bayesian Age-Period-Cohort (BAPC) model, are shown for males (A) and females (B). Shaded areas indicate the 95% uncertainty intervals. YLDs, years lived with disability. Data source: Global Burden of Disease Study 2021.

Supplementary file Table 1. Changes in YLDs cases according to population-level determinants and causes by SDI quintile from 1990 to 2021.				
Location	Over difference[a]	Change due to Population-level determinants (% contribute to the total changes)		
		Aging[b]	Population[c]	Epidemiological change[d]
Global	63091.26	29214.98 (46.31%)	127740.68 (202.47%)	-93864.39 (-148.78%)
High SDI	1901.07	3147.26 (165.55%)	6681.76 (351.47%)	-7927.95 (-417.03%)
High-middle SDI	17035.99	6292.05 (36.93%)	18210.22 (106.89%)	-7466.28 (-43.83%)
Middle SDI	26376.41	16019.10 (60.73%)	51048.78 (193.54%)	-40691.47 (-154.27%)
Low-middle SDI	14770.38	7673.02 (51.95%)	53808.63 (364.30%)	-46711.27 (-316.25%)
Low SDI	2987.06	-557.66 (-18.67%)	10857.12 (363.47%)	-7312.39 (-244.80%)

a.Change in YLDs cases between year 2021 and 1990
b.Change in YLDs cases due to change in the age structure
c.Change in YLDs cases due to change in population number
d.Change in YLDs cases due to epidemiologic changes. Epidemiologic changes refer to the YLDs cases change when age structure and population hold constant
SDI quintile includes high SDI, high-middle SDI, middle SDI, low-middle SDI, low SDI.
YLDs: Years Lived with Disability; SDI: Socio-demographic index.
Data source: Global Burden of Disease 2021.

Supplementary file Table 2. Estimated YLDs cases, age-standardized YLDs rate (per 100,000 population), and temporal trends of vision impairment due to smoking-related cataract by country or region, 1990–2021

	1990		2021		1990 to 2021
Characteristics	Cases (95% UI)	Age standardized YLDs rate per 100,000 population (95% UI)	Cases (95% UI)	Age standardized YLDs rate per 100,000 population (95% UI)	EAPC% (95% CI)
**Country**					
American Samoa	0.9 (0.6 to 1.3)	3.73 (2.47 to 5.37)	1.3 (0.9 to 2)	2.66 (1.74 to 3.94)	-1.12 (-1.14 to -1.1)
Antigua and Barbuda	0.5 (0.3 to 0.8)	0.97 (0.61 to 1.53)	0.7 (0.4 to 1.1)	0.65 (0.41 to 1.04)	-1.42 (-1.56 to -1.29)
Arab Republic of Egypt	1389 (918.3 to 1968.3)	5.74 (3.87 to 8.16)	2814.4 (1932.3 to 4069.2)	4.97 (3.44 to 7.16)	-0.52 (-0.62 to -0.42)
Argentine Republic	538.3 (361.1 to 798.2)	1.66 (1.11 to 2.48)	567.7 (364.5 to 852)	1.04 (0.67 to 1.56)	-1.42 (-1.51 to -1.34)
Australia	189.5 (127.9 to 270.5)	0.99 (0.67 to 1.41)	245 (159.7 to 368.7)	0.6 (0.39 to 0.91)	-1.5 (-1.57 to -1.43)
Barbados	1 (0.6 to 1.4)	0.32 (0.2 to 0.48)	1.1 (0.6 to 1.7)	0.22 (0.13 to 0.34)	-1.45 (-1.59 to -1.31)
Belize	1.3 (0.9 to 1.9)	1.44 (0.95 to 2.09)	2.4 (1.6 to 3.6)	0.83 (0.53 to 1.26)	-1.66 (-1.82 to -1.5)
Bermuda	0.6 (0.4 to 1)	1.01 (0.61 to 1.58)	1.1 (0.7 to 1.7)	0.83 (0.5 to 1.3)	-0.64 (-0.72 to -0.57)
Bolivarian Republic of Venezuela	239.5 (153.8 to 344.2)	2.49 (1.59 to 3.6)	289.5 (185.1 to 445.6)	0.96 (0.61 to 1.47)	-3.02 (-3.17 to -2.86)
Bosnia and Herzegovina	56.7 (38.1 to 81)	1.39 (0.93 to 1.99)	67.9 (44.6 to 100)	1.14 (0.76 to 1.67)	-0.63 (-0.94 to -0.31)
Brunei Darussalam	1.7 (1.1 to 2.5)	1.46 (0.98 to 2.12)	2.4 (1.5 to 3.6)	0.64 (0.41 to 0.94)	-2.64 (-2.78 to -2.51)
Burkina Faso	19.4 (12.4 to 29.2)	0.47 (0.3 to 0.72)	71.4 (45.9 to 107.2)	0.72 (0.45 to 1.11)	0.14 (-0.3 to 0.58)
Canada	414.2 (279.8 to 599.4)	1.29 (0.88 to 1.86)	518.4 (326.3 to 800.7)	0.76 (0.48 to 1.18)	-1.78 (-1.89 to -1.67)
Central African Republic	3.8 (2.4 to 5.5)	0.4 (0.25 to 0.58)	7.2 (4.4 to 10.8)	0.32 (0.2 to 0.47)	-0.68 (-0.93 to -0.42)
Commonwealth of Dominica	0.5 (0.3 to 0.7)	0.84 (0.53 to 1.27)	0.4 (0.3 to 0.7)	0.53 (0.32 to 0.84)	-1.45 (-1.71 to -1.2)
Commonwealth of the Bahamas	1.3 (0.8 to 2.1)	0.87 (0.53 to 1.36)	2.7 (1.5 to 4.3)	0.67 (0.39 to 1.1)	-0.84 (-0.94 to -0.74)
Cook Islands	0.7 (0.5 to 1)	5.42 (3.56 to 7.9)	0.8 (0.5 to 1.2)	3.06 (2 to 4.64)	-1.86 (-1.91 to -1.81)
Czech Republic	163 (109.1 to 235.9)	1.2 (0.8 to 1.74)	179 (119 to 266.4)	0.89 (0.59 to 1.32)	-0.97 (-1.04 to -0.9)
Democratic People's Republic of Korea	99.2 (62.9 to 146.2)	0.65 (0.42 to 0.96)	139.7 (84 to 219.7)	0.43 (0.26 to 0.67)	-1.63 (-1.71 to -1.55)
Democratic Republic of Sao Tome and Principe	0.8 (0.5 to 1.2)	1.17 (0.73 to 1.84)	0.9 (0.6 to 1.3)	0.74 (0.47 to 1.14)	-1.9 (-2.18 to -1.63)
Democratic Republic of Timor-Leste	16.3 (10.5 to 24)	6.46 (4.07 to 9.31)	25.3 (16.7 to 37.4)	3.15 (2.08 to 4.69)	-2.28 (-2.35 to -2.2)

Democratic Republic of the Congo	19.1 (12.6 to 28.3)	0.13 (0.08 to 0.19)	38.3 (24.6 to 57.4)	0.1 (0.06 to 0.15)	-0.69 (-0.81 to -0.57)
Democratic Socialist Republic of Sri Lanka	384.9 (260.1 to 548.9)	3.92 (2.67 to 5.61)	392.2 (265.9 to 580.7)	1.48 (1 to 2.21)	-3.31 (-3.49 to -3.14)
Dominican Republic	95 (60 to 142.5)	2.77 (1.74 to 4.15)	149.4 (92.6 to 223)	1.53 (0.94 to 2.29)	-1.91 (-2.03 to -1.78)
Eastern Republic of Uruguay	22 (14.4 to 32.1)	0.59 (0.39 to 0.87)	24.7 (15.5 to 37.8)	0.5 (0.32 to 0.75)	-0.65 (-0.74 to -0.57)
Federal Democratic Republic of Ethiopia	450.5 (301.8 to 627.2)	2.03 (1.37 to 2.8)	698.7 (480.1 to 979.3)	1.46 (1 to 2.07)	-0.74 (-0.88 to -0.6)
Federal Democratic Republic of Nepal	645.3 (457.8 to 877.8)	8 (5.58 to 10.85)	851.1 (583.2 to 1204.9)	3.9 (2.65 to 5.49)	-2.86 (-3.02 to -2.69)
Federal Republic of Germany	1552.9 (1042.9 to 2261.3)	1.29 (0.87 to 1.89)	1421.7 (905.6 to 2134.9)	0.83 (0.53 to 1.24)	-1.44 (-1.52 to -1.36)
Federal Republic of Nigeria	876.4 (595.5 to 1237)	1.99 (1.37 to 2.81)	1436.4 (964.9 to 2087.1)	1.49 (1 to 2.21)	-0.99 (-1.3 to -0.68)
Federal Republic of Somalia	43.5 (26.3 to 66.4)	1.61 (0.98 to 2.41)	75.5 (44.5 to 118.1)	1.07 (0.65 to 1.63)	-1.41 (-1.57 to -1.26)
Federated States of Micronesia	3 (2 to 4.2)	5.81 (3.89 to 8.29)	3.2 (2.1 to 4.6)	3.89 (2.57 to 5.69)	-1.37 (-1.4 to -1.34)
Federative Republic of Brazil	5307.1 (3582.7 to 7494.7)	6.09 (4.1 to 8.63)	6260.1 (4018.2 to 9439.2)	2.5 (1.61 to 3.77)	-2.64 (-2.95 to -2.33)
French Republic	963.7 (646.4 to 1396.8)	1.23 (0.82 to 1.8)	1006.5 (653.1 to 1503.7)	0.85 (0.56 to 1.27)	-1.19 (-1.29 to -1.1)
Gabonese Republic	3.6 (2.4 to 5.3)	0.62 (0.41 to 0.9)	4.6 (3 to 6.8)	0.41 (0.26 to 0.61)	-1.27 (-1.32 to -1.22)
Georgia	164.2 (108.5 to 236.2)	2.6 (1.73 to 3.71)	176 (117.1 to 255.9)	3.04 (2.05 to 4.38)	0.97 (0.82 to 1.13)
Grand Duchy of Luxembourg	6.3 (3.8 to 9.7)	1.21 (0.73 to 1.86)	9.2 (5.5 to 14.2)	0.9 (0.55 to 1.36)	-0.99 (-1.03 to -0.95)
Greenland	0.6 (0.4 to 0.9)	1.56 (1 to 2.38)	0.8 (0.5 to 1.1)	1.1 (0.7 to 1.6)	-1.28 (-1.33 to -1.24)
Grenada	0.7 (0.4 to 1.1)	1.02 (0.64 to 1.6)	0.6 (0.4 to 0.9)	0.49 (0.31 to 0.78)	-2.58 (-2.75 to -2.41)
Guam	2.5 (1.6 to 3.9)	2.94 (1.91 to 4.55)	5.3 (3.4 to 8.1)	2.49 (1.58 to 3.78)	-0.55 (-0.6 to -0.51)
Hashemite Kingdom of Jordan	51.8 (35.1 to 74.7)	4.38 (2.98 to 6.35)	166.2 (109.5 to 236)	2.37 (1.57 to 3.37)	-2.08 (-2.17 to -2)
Hellenic Republic	330.7 (223.5 to 480)	2.29 (1.57 to 3.32)	281.5 (187 to 417.9)	1.3 (0.87 to 1.93)	-1.86 (-1.94 to -1.78)
Hungary	334.7 (227.3 to 474.7)	2.35 (1.6 to 3.31)	211 (139 to 307.3)	1.22 (0.81 to 1.76)	-2.27 (-2.37 to -2.16)
Independent State of Papua New Guinea	122.7 (77.2 to 176.4)	6.13 (3.86 to 8.81)	280.8 (180.8 to 424.4)	4.85 (3.1 to 7.4)	-0.87 (-1.01 to -0.72)

Independent State of Samoa	5.3 (3.6 to 7.4)	6.05 (4.13 to 8.46)	6.4 (4.3 to 9.5)	4.38 (2.93 to 6.49)	-1.14 (-1.21 to -1.06)
Ireland	73.1 (50.3 to 105.2)	1.85 (1.28 to 2.68)	64.5 (41.7 to 95.8)	0.84 (0.55 to 1.25)	-2.91 (-3.04 to -2.78)
Islamic Republic of Afghanistan	297.6 (181.4 to 466.6)	4.61 (2.81 to 7.28)	315.8 (205.4 to 463.3)	3.39 (2.1 to 5.24)	-0.42 (-0.9 to 0.06)
Islamic Republic of Iran	844.3 (568.3 to 1249.9)	3.2 (2.18 to 4.69)	1995 (1352.9 to 2971.7)	2.55 (1.74 to 3.82)	-0.75 (-0.83 to -0.67)
Islamic Republic of Mauritania	17.3 (12.2 to 23.9)	1.62 (1.13 to 2.25)	21.2 (14.4 to 30.1)	0.91 (0.62 to 1.3)	-1.9 (-1.93 to -1.87)
Islamic Republic of Pakistan	9761.3 (6922.4 to 13356.4)	17.83 (12.63 to 24.44)	11369.2 (7910.6 to 15793.1)	9.65 (6.73 to 13.33)	-2.39 (-2.65 to -2.14)
Jamaica	27.1 (18.1 to 40)	1.54 (1.02 to 2.25)	26.7 (16.9 to 42)	0.87 (0.55 to 1.34)	-2.09 (-2.18 to -2)
Japan	2052.2 (1451.8 to 2886.9)	1.22 (0.87 to 1.72)	2050.4 (1354.9 to 3014.8)	0.69 (0.47 to 0.97)	-2 (-2.06 to -1.93)
Kingdom of Bahrain	11 (6.9 to 16.8)	6.31 (4 to 9.81)	34.6 (21.3 to 53.9)	3.72 (2.35 to 5.85)	-1.89 (-1.99 to -1.79)
Kingdom of Belgium	232.1 (157.3 to 344)	1.61 (1.09 to 2.38)	220.6 (143.2 to 335.4)	1.05 (0.68 to 1.57)	-1.32 (-1.38 to -1.26)
Kingdom of Bhutan	4.3 (2.4 to 6.6)	2.05 (1.15 to 3.2)	5.7 (3.6 to 8.6)	0.99 (0.61 to 1.5)	-2.6 (-2.69 to -2.52)
Kingdom of Cambodia	672.4 (475.4 to 944.9)	15.66 (11.19 to 21.83)	1039.6 (698.1 to 1471.5)	8.84 (5.93 to 12.47)	-2.31 (-2.56 to -2.05)
Kingdom of Denmark	178.1 (118.5 to 253.8)	2.24 (1.5 to 3.13)	158.8 (102.7 to 243.6)	1.38 (0.91 to 2.1)	-1.63 (-1.75 to -1.51)
Kingdom of Eswatini	6.4 (4.2 to 9)	2.42 (1.58 to 3.37)	5.5 (3.5 to 8.4)	1.09 (0.69 to 1.62)	-2.68 (-2.74 to -2.62)
Kingdom of Lesotho	35 (22.6 to 49.5)	4.32 (2.77 to 6.32)	35.1 (22.9 to 50.9)	3.41 (2.25 to 5.05)	-0.58 (-0.66 to -0.51)
Kingdom of Morocco	523.3 (352.5 to 738.7)	3.65 (2.45 to 5.12)	617.7 (411 to 909.6)	1.74 (1.15 to 2.61)	-2.48 (-2.64 to -2.33)
Kingdom of Norway	66.9 (46.2 to 97.7)	1.07 (0.75 to 1.57)	56.8 (38.4 to 83.9)	0.63 (0.42 to 0.94)	-1.97 (-2.11 to -1.82)
Kingdom of Saudi Arabia	562.8 (361.1 to 841.3)	9.1 (5.82 to 13.62)	999.4 (635 to 1465.1)	4.5 (2.81 to 6.68)	-2.3 (-2.43 to -2.16)
Kingdom of Spain	1681.4 (1138.4 to 2407.1)	3.34 (2.28 to 4.79)	1569.1 (1039.6 to 2354.1)	1.84 (1.22 to 2.71)	-1.91 (-1.96 to -1.86)
Kingdom of Sweden	111.9 (74.6 to 165.1)	0.78 (0.53 to 1.16)	149.9 (93.1 to 238.7)	0.74 (0.46 to 1.14)	-0.15 (-0.27 to -0.03)
Kingdom of Thailand	2162.1 (1540.3 to 2996.7)	6.44 (4.59 to 8.9)	3166.5 (2133 to 4422.7)	2.91 (1.96 to 4.06)	-2.71 (-2.85 to -2.56)
Kingdom of Tonga	2 (1.3 to 2.8)	3.64 (2.37 to 5.19)	1.7 (1.1 to 2.6)	2.21 (1.44 to 3.26)	-1.61 (-1.64 to -1.58)
Kingdom of the Netherlands	204.3 (137.3 to 303.7)	1.06 (0.72 to 1.58)	238 (152.5 to 360.1)	0.72 (0.47 to 1.1)	-0.94 (-1.05 to -0.83)
Kyrgyz Republic	78.6 (55 to 111.6)	2.63 (1.85 to 3.78)	167.5 (112.2 to 235.1)	3.44 (2.28 to 4.85)	1.77 (1.47 to 2.07)
Lao People's Democratic Republic	37 (24.8 to 53.4)	1.99 (1.33 to 2.83)	62 (40.9 to 90.1)	1.5 (1.01 to 2.19)	-0.85 (-0.89 to -0.81)
Lebanese Republic	255 (165.4 to 359.6)	12.04 (7.84 to 16.97)	498.8 (333.7 to 721.6)	8.27 (5.54 to 11.89)	-1.34 (-1.54 to -1.13)
Malaysia	830.7 (592.5 to 1166.5)	9.07 (6.43 to 12.65)	1028.6 (726.2 to 1414)	3.73 (2.6 to 5.1)	-3.07 (-3.18 to -2.95)

Mongolia	20 (13.1 to 28.3)	1.88 (1.25 to 2.63)	38.8 (26 to 54.9)	1.63 (1.11 to 2.31)	-0.11 (-0.24 to 0.03)
Montenegro	9.2 (5.9 to 14.2)	1.46 (0.93 to 2.24)	11.6 (7.4 to 17.5)	1.19 (0.76 to 1.77)	-0.85 (-1.1 to -0.6)
New Zealand	49.2 (32.7 to 71.3)	1.28 (0.85 to 1.86)	60.6 (38.7 to 94.6)	0.75 (0.48 to 1.16)	-1.71 (-1.84 to -1.57)
North Macedonia	29.9 (19.5 to 44)	1.59 (1.04 to 2.32)	36.2 (23.8 to 52.4)	1.12 (0.74 to 1.62)	-1.32 (-1.39 to -1.25)
Northern Mariana Islands	0.7 (0.4 to 1)	3.6 (2.38 to 5.17)	1.4 (0.9 to 2.2)	2.58 (1.61 to 3.81)	-1.09 (-1.13 to -1.05)
Palestine	81.8 (55 to 121.8)	9.75 (6.54 to 14.49)	144.1 (97 to 214.2)	5.73 (3.85 to 8.48)	-1.77 (-1.85 to -1.69)
People's Democratic Republic of Algeria	938.8 (649 to 1337.3)	8.42 (5.78 to 12.03)	1561.1 (1062.5 to 2207.3)	4.7 (3.26 to 6.69)	-1.92 (-2.07 to -1.78)
People's Republic of Bangladesh	4296.5 (3009.6 to 5873)	9.89 (6.92 to 13.49)	8228.2 (5570.4 to 11770.5)	6.09 (4.15 to 8.68)	-1.08 (-1.27 to -0.88)
People's Republic of China	31757.7 (22344 to 44078)	3.95 (2.79 to 5.45)	60057.8 (41169.6 to 84681.4)	2.83 (1.94 to 4)	-0.61 (-0.84 to -0.37)
Plurinational State of Bolivia	106.6 (70.8 to 155.3)	3.4 (2.25 to 4.99)	164.2 (107.6 to 238.4)	1.81 (1.18 to 2.64)	-1.83 (-2.01 to -1.64)
Portuguese Republic	123.8 (82.7 to 179.7)	0.94 (0.63 to 1.37)	102.9 (66.6 to 155.2)	0.53 (0.35 to 0.79)	-2.02 (-2.13 to -1.9)
Principality of Andorra	0.7 (0.4 to 1.1)	1.2 (0.77 to 1.89)	1.4 (0.8 to 2.1)	0.91 (0.56 to 1.39)	-0.92 (-0.97 to -0.87)
Principality of Monaco	0.8 (0.5 to 1.2)	1.25 (0.77 to 1.97)	0.8 (0.5 to 1.3)	0.95 (0.56 to 1.56)	-0.93 (-0.96 to -0.89)
Puerto Rico	41.8 (25.1 to 67.4)	1.15 (0.69 to 1.84)	53.7 (32 to 92.3)	0.77 (0.45 to 1.27)	-1.44 (-1.5 to -1.37)
Republic of Albania	34.9 (23.6 to 50.3)	1.75 (1.18 to 2.52)	66.6 (43.8 to 98.6)	1.55 (1.02 to 2.28)	-0.36 (-0.43 to -0.3)
Republic of Angola	74.7 (47.6 to 107.9)	1.8 (1.17 to 2.56)	126.9 (83.6 to 183.9)	0.98 (0.65 to 1.42)	-1.85 (-1.94 to -1.76)
Republic of Armenia	99 (66.2 to 141.6)	3.59 (2.4 to 5.04)	141.5 (94.3 to 203)	3.26 (2.18 to 4.67)	-0.17 (-0.23 to -0.12)
Republic of Austria	102.3 (68.8 to 153.4)	0.96 (0.65 to 1.4)	173.5 (113.7 to 257)	1.09 (0.72 to 1.59)	0.51 (0.28 to 0.73)
Republic of Azerbaijan	151.1 (98.8 to 222.2)	2.96 (1.92 to 4.34)	319.1 (210.8 to 457.3)	3.16 (2.08 to 4.47)	0.42 (0.15 to 0.68)
Republic of Belarus	200.2 (131.1 to 292.1)	1.57 (1.04 to 2.29)	199.4 (134.8 to 289.7)	1.28 (0.87 to 1.85)	-0.55 (-0.61 to -0.5)
Republic of Benin	19.8 (13.1 to 29)	1.02 (0.67 to 1.5)	50.6 (32.7 to 76.2)	0.96 (0.61 to 1.44)	-0.52 (-0.89 to -0.15)
Republic of Botswana	23.3 (15.8 to 33.5)	4.2 (2.87 to 5.99)	31.2 (21.2 to 44.5)	2.09 (1.41 to 2.95)	-2.27 (-2.36 to -2.18)
Republic of Bulgaria	89.3 (59.3 to 129.8)	0.74 (0.49 to 1.09)	62.3 (40.1 to 92.5)	0.5 (0.32 to 0.75)	-1.23 (-1.41 to -1.05)
Republic of Burundi	13.5 (9 to 19.2)	0.57 (0.39 to 0.82)	14.6 (9.1 to 22.6)	0.28 (0.18 to 0.43)	-2.55 (-3.01 to -2.1)
Republic of Cabo Verde	3.6 (2.4 to 5.1)	1.62 (1.08 to 2.3)	3 (1.9 to 4.4)	0.64 (0.41 to 0.95)	-3.09 (-3.25 to -2.94)
Republic of Cameroon	38.4 (25.7 to 57.4)	0.89 (0.61 to 1.33)	74.8 (48.1 to 111.8)	0.58 (0.37 to 0.87)	-1.39 (-1.5 to -1.27)
Republic of Chad	42.6 (25.8 to 61.3)	1.53 (0.94 to 2.21)	69.8 (44.4 to 103.3)	1.25 (0.8 to 1.88)	-0.79 (-0.87 to -0.71)

Republic of Chile	222.5 (150.1 to 319.3)	2.08 (1.39 to 3.01)	277.8 (181 to 410.5)	1.14 (0.74 to 1.67)	-2.08 (-2.13 to -2.03)
Republic of Colombia	393.2 (260.6 to 560.4)	2.22 (1.47 to 3.15)	497.1 (316.5 to 742.6)	0.9 (0.57 to 1.34)	-3 (-3.1 to -2.89)
Republic of Costa Rica	54.8 (36.5 to 79.5)	3.17 (2.11 to 4.61)	92.6 (60.2 to 137.8)	1.69 (1.09 to 2.51)	-2 (-2.09 to -1.91)
Republic of Croatia	82.1 (56.1 to 117.2)	1.37 (0.93 to 1.95)	83.5 (54 to 121.4)	1 (0.66 to 1.46)	-0.74 (-0.85 to -0.64)
Republic of Cuba	423 (287.7 to 617.9)	4.14 (2.81 to 6.02)	402.7 (264.4 to 606.6)	2.09 (1.38 to 3.15)	-2.36 (-2.49 to -2.23)
Republic of Cyprus	10.8 (7.2 to 15.4)	1.35 (0.89 to 1.92)	22 (14.6 to 32.4)	1.1 (0.73 to 1.62)	-0.59 (-0.65 to -0.53)
Republic of C么te d'Ivoire	28.4 (19 to 41.2)	0.81 (0.53 to 1.18)	152.3 (102.2 to 232)	1.29 (0.86 to 1.98)	-0.53 (-1.4 to 0.34)
Republic of Djibouti	5.1 (3.2 to 7.6)	3.64 (2.35 to 5.35)	13.3 (8.5 to 19.6)	2.06 (1.32 to 3.02)	-1.75 (-1.78 to -1.73)
Republic of Ecuador	147.2 (96.7 to 213.4)	2.87 (1.86 to 4.14)	188.4 (121 to 278.4)	1.15 (0.74 to 1.7)	-2.99 (-3.12 to -2.86)
Republic of El Salvador	52.3 (31.1 to 82)	1.76 (1.04 to 2.79)	64.4 (38 to 100.2)	1.04 (0.62 to 1.62)	-1.48 (-1.58 to -1.39)
Republic of Equatorial Guinea	8.6 (5.1 to 13.4)	4.27 (2.5 to 6.52)	7.1 (4.2 to 11)	1.18 (0.71 to 1.83)	-4.5 (-4.7 to -4.3)
Republic of Estonia	17.7 (11.4 to 25.8)	0.87 (0.56 to 1.27)	18.4 (12 to 27.3)	0.77 (0.5 to 1.13)	-0.44 (-0.63 to -0.24)
Republic of Fiji	21.1 (14.4 to 29.1)	5.48 (3.7 to 7.62)	25.8 (17.8 to 36.8)	3.13 (2.14 to 4.52)	-1.65 (-1.73 to -1.57)
Republic of Finland	52.3 (35.4 to 77.3)	0.79 (0.53 to 1.15)	62.1 (38.6 to 96.7)	0.6 (0.38 to 0.91)	-0.63 (-0.75 to -0.51)
Republic of Ghana	38.6 (25.4 to 57.2)	0.76 (0.5 to 1.14)	136.4 (85.5 to 208.5)	0.89 (0.56 to 1.37)	0.58 (0.49 to 0.67)
Republic of Guatemala	61.9 (39.8 to 92.5)	1.97 (1.27 to 2.91)	110.5 (69.6 to 163.4)	1.03 (0.65 to 1.53)	-2.05 (-2.09 to -2.02)
Republic of Guinea	53.7 (35 to 74.9)	1.67 (1.09 to 2.35)	83.2 (54.7 to 116.5)	1.47 (0.97 to 2.1)	-0.52 (-0.67 to -0.37)
Republic of Guinea-Bissau	1.9 (1.2 to 2.9)	0.49 (0.31 to 0.76)	4.2 (2.7 to 6.4)	0.55 (0.34 to 0.84)	0.47 (0.38 to 0.55)
Republic of Guyana	4.9 (3.1 to 7.1)	1.26 (0.8 to 1.88)	4.2 (2.7 to 6.1)	0.66 (0.42 to 0.97)	-1.91 (-2.04 to -1.77)
Republic of Haiti	38.5 (23.9 to 58.5)	1.15 (0.7 to 1.75)	41.6 (25.5 to 62.3)	0.57 (0.34 to 0.91)	-2.34 (-2.51 to -2.18)
Republic of Honduras	38.2 (25.2 to 55)	1.91 (1.25 to 2.76)	63.4 (41.7 to 96.9)	1.02 (0.67 to 1.55)	-2.1 (-2.21 to -2)
Republic of Iceland	4.3 (2.8 to 6.6)	1.57 (1.02 to 2.36)	5.4 (3.4 to 8.4)	0.99 (0.64 to 1.54)	-1.51 (-1.62 to -1.39)
Republic of India	47793.4 (33912.2 to 65346.5)	11.28 (8.01 to 15.39)	59155 (42040.9 to 82710.5)	5.21 (3.69 to 7.35)	-2.44 (-2.54 to -2.33)
Republic of Indonesia	7285 (5163.7 to 9974.2)	7.7 (5.47 to 10.54)	13487.5 (9538 to 18757)	5.72 (4.06 to 7.81)	-1.14 (-1.34 to -0.94)
Republic of Iraq	851.3 (588.3 to 1188.5)	11.31 (7.8 to 15.94)	1111.9 (755.5 to 1633)	5.07 (3.44 to 7.55)	-2.67 (-2.72 to -2.62)
Republic of Italy	2538.3 (1744.3 to 3570.8)	3.09 (2.14 to 4.34)	2078.2 (1400.6 to 3071)	1.68 (1.14 to 2.46)	-1.97 (-2.06 to -1.89)
Republic of Kazakhstan	264.8 (175.8 to 386.2)	2 (1.33 to 2.92)	298.3 (195.7 to 432.1)	1.56 (1.03 to 2.26)	-0.74 (-0.88 to -0.59)
Republic of Kenya	371.7 (260.5 to 526.3)	4.32 (3.02 to 6.09)	414 (285.4 to 583.3)	1.68 (1.16 to 2.38)	-3.23 (-3.51 to -2.95)

Republic of Kiribati	3.1 (2.2 to 4.4)	8.26 (5.82 to 11.65)	5.2 (3.6 to 7.3)	7.08 (4.89 to 9.85)	-0.66 (-0.96 to -0.36)
Republic of Korea	496.8 (327.9 to 732)	1.6 (1.06 to 2.29)	640.7 (422.4 to 931.5)	0.72 (0.48 to 1.03)	-2.79 (-2.9 to -2.68)
Republic of Latvia	45.8 (31.5 to 65.7)	1.3 (0.89 to 1.86)	31.4 (20.8 to 46.1)	0.9 (0.61 to 1.31)	-1.08 (-1.16 to -0.99)
Republic of Liberia	15.4 (10.1 to 22.6)	1.32 (0.88 to 1.95)	16.5 (10.8 to 23.5)	0.72 (0.46 to 1.05)	-2.22 (-2.34 to -2.1)
Republic of Lithuania	57.5 (38.6 to 82.5)	1.29 (0.87 to 1.86)	47 (30 to 67.7)	0.89 (0.58 to 1.29)	-1.24 (-1.27 to -1.21)
Republic of Madagascar	50.1 (34 to 70.5)	1.03 (0.7 to 1.47)	40.2 (27.2 to 56.7)	0.36 (0.25 to 0.52)	-3.55 (-3.81 to -3.28)
Republic of Malawi	105.3 (69.1 to 149.5)	2.88 (1.89 to 4.07)	126.5 (81.8 to 180)	1.72 (1.1 to 2.45)	-1.51 (-1.58 to -1.44)
Republic of Maldives	3.1 (2.1 to 4.4)	4.4 (2.97 to 6.25)	5 (3.4 to 7.3)	1.59 (1.06 to 2.31)	-3.56 (-3.82 to -3.3)
Republic of Mali	66.2 (44.2 to 93.7)	1.74 (1.16 to 2.51)	210.4 (143.1 to 294.1)	2.45 (1.65 to 3.47)	1.08 (0.9 to 1.26)
Republic of Malta	5.8 (3.8 to 8.4)	1.38 (0.91 to 1.99)	6.7 (4.4 to 10.2)	0.83 (0.54 to 1.24)	-1.82 (-1.9 to -1.74)
Republic of Mauritius	28.6 (19.8 to 40.7)	4.02 (2.8 to 5.73)	50.1 (33.1 to 73.1)	2.73 (1.81 to 3.97)	-1.21 (-1.6 to -0.82)
Republic of Moldova	144.8 (94.6 to 212.8)	3.23 (2.1 to 4.76)	177.7 (111.3 to 263.7)	3.06 (1.93 to 4.54)	0.02 (-0.12 to 0.16)
Republic of Mozambique	129.8 (86.1 to 181.1)	2.17 (1.46 to 3.09)	148.2 (98.7 to 218.7)	1.3 (0.87 to 1.89)	-1.48 (-1.58 to -1.38)
Republic of Namibia	23.8 (15.8 to 33.5)	4.23 (2.86 to 5.98)	23.7 (15.9 to 34)	1.95 (1.29 to 2.77)	-2.74 (-2.86 to -2.62)
Republic of Nauru	0.3 (0.2 to 0.4)	5.3 (3.57 to 7.91)	0.2 (0.1 to 0.3)	3.4 (2.27 to 4.96)	-1.48 (-1.62 to -1.34)
Republic of Nicaragua	42.2 (27.5 to 61.8)	2.77 (1.8 to 4.12)	72.2 (47.2 to 104.8)	1.49 (0.96 to 2.18)	-1.74 (-1.96 to -1.53)
Republic of Niue	0.1 (0 to 0.1)	3.54 (2.23 to 5.38)	0.1 (0 to 0.1)	2.42 (1.49 to 3.69)	-1.32 (-1.37 to -1.27)
Republic of Palau	0.3 (0.2 to 0.5)	3.34 (2.07 to 5.16)	0.6 (0.4 to 0.9)	2.42 (1.53 to 3.64)	-1.12 (-1.17 to -1.07)
Republic of Panama	65.1 (44.2 to 91.5)	4.47 (2.99 to 6.27)	91.3 (57.6 to 140.4)	2.06 (1.3 to 3.17)	-2.61 (-2.72 to -2.5)
Republic of Paraguay	149.9 (102.7 to 209.7)	7.09 (4.81 to 10.02)	148.1 (94.4 to 223.8)	2.64 (1.69 to 4.01)	-3.46 (-3.61 to -3.31)
Republic of Peru	345.5 (221.6 to 521.1)	3.02 (1.92 to 4.62)	660.5 (401.7 to 1040.4)	1.99 (1.2 to 3.14)	-2.14 (-2.42 to -1.86)
Republic of Poland	635.6 (437.4 to 904.9)	1.46 (1.01 to 2.09)	530.2 (358.6 to 765.8)	0.79 (0.54 to 1.13)	-2.15 (-2.22 to -2.09)
Republic of Rwanda	42.4 (27.9 to 61.3)	1.69 (1.11 to 2.44)	63 (38.5 to 95.1)	1.13 (0.68 to 1.71)	-1.41 (-1.51 to -1.31)
Republic of San Marino	0.4 (0.2 to 0.6)	1.22 (0.77 to 1.83)	0.6 (0.4 to 1)	0.92 (0.56 to 1.46)	-0.96 (-1.03 to -0.9)
Republic of Senegal	47.3 (31.1 to 67.4)	1.35 (0.88 to 1.93)	57.1 (37 to 87.6)	0.69 (0.43 to 1.08)	-2.07 (-2.16 to -1.98)
Republic of Serbia	131.5 (86.8 to 193.2)	1.15 (0.76 to 1.68)	132.6 (85 to 197.4)	0.87 (0.56 to 1.29)	-1.1 (-1.48 to -0.72)
Republic of Seychelles	2.7 (1.8 to 3.8)	4.8 (3.26 to 6.73)	3.5 (2.3 to 5)	3.06 (2.06 to 4.5)	-1.56 (-1.66 to -1.47)
Republic of Sierra Leone	17.6 (11.5 to 25)	0.87 (0.57 to 1.23)	20.6 (13.8 to 29.8)	0.51 (0.34 to 0.74)	-1.77 (-1.98 to -1.56)
Republic of Singapore	19.5 (13 to 28.1)	0.81 (0.55 to 1.17)	38.9 (25.5 to 57.4)	0.45 (0.29 to 0.66)	-1.92 (-2.07 to -1.78)

Republic of Slovenia	25.1 (16.1 to 38.4)	1.03 (0.66 to 1.58)	33 (20.4 to 51.4)	0.84 (0.53 to 1.31)	-0.36 (-0.49 to -0.23)
Republic of South Africa	1110.7 (762.9 to 1611.1)	5.29 (3.61 to 7.58)	715.5 (488.7 to 990.1)	1.48 (1.01 to 2.05)	-4.5 (-4.78 to -4.22)
Republic of South Sudan	111.7 (67 to 167.1)	4.48 (2.69 to 6.67)	84 (54 to 128.6)	2.37 (1.49 to 3.74)	-2.17 (-2.26 to -2.08)
Republic of Sudan	462.6 (287.8 to 683.5)	5.22 (3.22 to 7.8)	493.9 (313.8 to 737)	2.72 (1.71 to 4.1)	-2.08 (-2.2 to -1.96)
Republic of Suriname	8.6 (5.7 to 12.3)	3.29 (2.21 to 4.74)	9.9 (6.4 to 14.9)	1.55 (1 to 2.34)	-2.85 (-3.01 to -2.68)
Republic of Tajikistan	102.7 (68.8 to 146)	3.77 (2.55 to 5.37)	108.6 (67.5 to 160.4)	1.85 (1.14 to 2.8)	-2.18 (-2.29 to -2.06)
Republic of Trinidad and Tobago	10.7 (7.1 to 15.9)	1.26 (0.84 to 1.87)	12.3 (7.8 to 18.8)	0.64 (0.4 to 0.97)	-2.49 (-2.64 to -2.33)
Republic of Tunisia	411.7 (269.9 to 571.5)	8.66 (5.67 to 12.04)	502.4 (346.8 to 705.2)	3.84 (2.66 to 5.39)	-2.66 (-2.77 to -2.56)
Republic of Turkey	2062.2 (1420.2 to 2931.7)	6 (4.12 to 8.47)	2234.4 (1463.7 to 3171.5)	2.36 (1.54 to 3.32)	-3.33 (-3.49 to -3.16)
Republic of Uganda	43.5 (29.5 to 61.3)	0.72 (0.48 to 1.03)	78.8 (50.1 to 116.9)	0.55 (0.35 to 0.81)	-1.04 (-1.26 to -0.82)
Republic of Uzbekistan	147.5 (97.4 to 215.9)	1.26 (0.83 to 1.84)	367.4 (244.1 to 521.1)	1.34 (0.89 to 1.94)	0.12 (-0.22 to 0.46)
Republic of Vanuatu	1.6 (1.1 to 2.3)	2.54 (1.71 to 3.59)	2.7 (1.8 to 3.8)	1.53 (1.03 to 2.21)	-1.83 (-1.98 to -1.68)
Republic of Yemen	268 (176.2 to 375.7)	5.54 (3.67 to 7.8)	679.1 (456.2 to 965.6)	4.93 (3.27 to 6.96)	-0.8 (-1.05 to -0.55)
Republic of Zambia	67.7 (46.3 to 95.4)	2.53 (1.7 to 3.61)	85.1 (56.9 to 121.7)	1.3 (0.86 to 1.85)	-2.4 (-2.52 to -2.28)
Republic of Zimbabwe	187.2 (125.2 to 271.5)	4.83 (3.16 to 6.94)	190.3 (121.6 to 281.1)	2.91 (1.85 to 4.38)	-1.71 (-1.8 to -1.61)
Republic of the Congo	8.8 (5.7 to 12.5)	0.82 (0.53 to 1.18)	18.5 (11.6 to 27)	0.68 (0.43 to 0.99)	-0.33 (-0.51 to -0.14)
Republic of the Gambia	15.8 (10.8 to 22.4)	4.1 (2.77 to 5.81)	17.7 (12 to 25.5)	1.73 (1.15 to 2.48)	-2.9 (-3.02 to -2.79)
Republic of the Marshall Islands	0.6 (0.4 to 0.9)	3.62 (2.26 to 5.48)	0.9 (0.6 to 1.3)	2.47 (1.56 to 3.87)	-1.22 (-1.23 to -1.21)
Republic of the Niger	23.9 (15.8 to 33.7)	0.86 (0.56 to 1.21)	68.5 (44.5 to 99.7)	0.87 (0.57 to 1.26)	-0.01 (-0.09 to 0.06)
Republic of the Philippines	2429.7 (1689.5 to 3358.6)	8.56 (5.88 to 11.7)	3346.3 (2294.2 to 4689.2)	4.18 (2.88 to 5.85)	-2.31 (-2.41 to -2.22)
Republic of the Union of Myanmar	3708.4 (2516.2 to 5082)	17.49 (11.77 to 23.96)	2406.8 (1622.8 to 3355.9)	5.18 (3.54 to 7.15)	-4.33 (-4.48 to -4.18)
Romania	262.4 (175.5 to 377.1)	0.95 (0.64 to 1.38)	200.7 (133.6 to 289.9)	0.6 (0.4 to 0.88)	-1.76 (-1.87 to -1.64)
Russian Federation	1564.3 (1091.1 to 2225.5)	0.87 (0.61 to 1.24)	2150.8 (1492.4 to 3093.8)	0.94 (0.66 to 1.35)	0.27 (-0.11 to 0.65)
Saint Kitts and Nevis	0.3 (0.2 to 0.5)	0.82 (0.5 to 1.31)	0.3 (0.2 to 0.4)	0.42 (0.26 to 0.69)	-2.33 (-2.44 to -2.23)
Saint Lucia	1.1 (0.7 to 1.6)	1.23 (0.8 to 1.85)	1.4 (0.9 to 2.2)	0.58 (0.36 to 0.92)	-2.6 (-2.71 to -2.49)
Saint Vincent and the Grenadines	0.6 (0.4 to 1)	0.89 (0.56 to 1.37)	0.9 (0.6 to 1.4)	0.65 (0.4 to 1.01)	-1.08 (-1.15 to -1.01)
Slovak Republic	52.3 (33.9 to 77.2)	0.89 (0.58 to 1.31)	62.1 (40.5 to 93.5)	0.68 (0.44 to 1.02)	-0.76 (-0.98 to -0.55)

Socialist Republic of Viet Nam	1933.3 (1336.3 to 2736.3)	4.98 (3.46 to 7.01)	2705.8 (1878 to 3844.1)	2.81 (1.97 to 4)	-2.22 (-2.34 to -2.09)
Solomon Islands	10.1 (6.9 to 14.3)	7.05 (4.85 to 10.01)	18.9 (13.1 to 26.5)	5.23 (3.65 to 7.4)	-0.73 (-0.86 to -0.61)
State of Eritrea	17.9 (11.1 to 28.7)	1.25 (0.79 to 2)	21.5 (13.8 to 33.8)	0.63 (0.41 to 1)	-2.2 (-2.32 to -2.09)
State of Israel	59.6 (40.3 to 88.9)	1.28 (0.85 to 1.9)	87.6 (57.7 to 131.6)	0.75 (0.5 to 1.12)	-1.74 (-1.79 to -1.69)
State of Kuwait	36.6 (24.4 to 53.1)	6.01 (4.02 to 8.62)	113.9 (75.4 to 164.3)	3.97 (2.6 to 5.79)	-1.43 (-1.51 to -1.36)
State of Libya	142.7 (97 to 203.1)	7.54 (5.12 to 10.67)	211.2 (146.6 to 296.5)	4 (2.75 to 5.52)	-2.05 (-2.23 to -1.86)
State of Qatar	6.3 (3.8 to 9.6)	4.32 (2.7 to 6.56)	33.6 (20.8 to 51.5)	2.51 (1.54 to 3.76)	-1.83 (-1.96 to -1.7)
Sultanate of Oman	51.1 (34.8 to 71.9)	6.69 (4.52 to 9.42)	87.9 (58.7 to 126.1)	3.7 (2.43 to 5.29)	-2.05 (-2.12 to -1.98)
Swiss Confederation	130 (88.7 to 188.3)	1.31 (0.89 to 1.89)	149.6 (98.1 to 220.4)	0.87 (0.58 to 1.29)	-1.26 (-1.34 to -1.17)
Syrian Arab Republic	461.5 (316.9 to 637.7)	9.35 (6.4 to 13.06)	522.4 (356.6 to 754.6)	4.1 (2.82 to 5.84)	-2.8 (-2.93 to -2.67)
Taiwan (Province of China)	56.4 (33.9 to 90.2)	0.39 (0.24 to 0.62)	103.2 (58.8 to 162.6)	0.24 (0.14 to 0.38)	-1.71 (-1.79 to -1.64)
Togolese Republic	31.6 (21 to 46.2)	2.74 (1.83 to 3.98)	75.4 (47.5 to 116.3)	1.91 (1.22 to 2.93)	-1.27 (-1.32 to -1.22)
Tokelau	0.1 (0 to 0.1)	4.61 (2.95 to 6.91)	0 (0 to 0.1)	2.83 (1.89 to 4.19)	-1.7 (-1.76 to -1.64)
Turkmenistan	123.2 (82.5 to 176.7)	6.22 (4.19 to 8.97)	154.4 (99.6 to 230.6)	3.63 (2.34 to 5.37)	-1.91 (-2.05 to -1.76)
Tuvalu	0.4 (0.2 to 0.5)	5.08 (3.35 to 7.16)	0.4 (0.2 to 0.6)	3.43 (2.23 to 5.13)	-1.28 (-1.3 to -1.25)
Ukraine	966.2 (666.2 to 1392.2)	1.38 (0.94 to 1.96)	767 (529.1 to 1093.6)	1.04 (0.72 to 1.48)	-0.94 (-1.18 to -0.71)
Union of the Comoros	4.6 (2.9 to 6.9)	2.42 (1.56 to 3.62)	5.9 (3.8 to 8.5)	1.23 (0.8 to 1.81)	-2.31 (-2.38 to -2.24)
United Arab Emirates	22.5 (15.2 to 32.1)	4.44 (2.97 to 6.43)	163.3 (102.8 to 246.1)	3.85 (2.44 to 5.82)	-0.45 (-0.66 to -0.23)
United Kingdom of Great Britain and Northern Ireland	1510.3 (1004.7 to 2191.6)	1.76 (1.17 to 2.56)	1063.8 (677.1 to 1639.9)	0.85 (0.55 to 1.3)	-2.71 (-2.88 to -2.53)
United Mexican States	1599.8 (1107.9 to 2218.6)	4.02 (2.79 to 5.53)	1502.2 (1027 to 2171.7)	1.22 (0.83 to 1.77)	-4.26 (-4.41 to -4.11)
United Republic of Tanzania	333.6 (225.9 to 484.2)	3.18 (2.14 to 4.57)	531.6 (330 to 799.9)	2 (1.23 to 3.07)	-1.46 (-1.58 to -1.34)
United States Virgin Islands	0.8 (0.5 to 1.4)	0.98 (0.55 to 1.64)	1.3 (0.7 to 2.2)	0.71 (0.41 to 1.18)	-1.03 (-1.08 to -0.98)
United States of America	4241.2 (2885.3 to 6183.3)	1.37 (0.94 to 1.99)	4467.5 (2955.3 to 6804.1)	0.81 (0.54 to 1.21)	-1.88 (-2.06 to -1.7)

YLDs: years lived with disability. UI: uncertainty intervals. CI: confidence interval. EAPC: estimated annual percentage change. Data source: Global Burden of Disease 2021.

© **2025 Chen Y. et al.**
